# Preoperative inflammatory biomarkers reveal renal involvement in postsurgical mortality in hip fracture patients: an exploratory study

**DOI:** 10.3389/fimmu.2024.1372079

**Published:** 2024-06-10

**Authors:** Ana M. Valdes, Adeel Ikram, Lauren A. Taylor, Amy Zheng, Afroditi Kouraki, Anthony Kelly, Waheed Ashraf, Amrita Vijay, Suzanne Miller, Jessica Nightingale, Nicholas M. Selby, Benjamin J. Ollivere

**Affiliations:** ^1^ Injury, Recovery and Inflammation Sciences, School of Medicine, University of Nottingham, Nottingham, United Kingdom; ^2^ National Institute for Health and Care Research (NIHR) Nottingham Biomedical Research Centre, Nottingham, United Kingdom; ^3^ Centre for Kidney Research and Innovation, Academic Unit for Translational Medical Sciences, School of Medicine, University of Nottingham, Nottingham, United Kingdom

**Keywords:** hip fracture, frailty, inflammation, FGF23, renal, surgery, acute kidney injury, mortality

## Abstract

**Background:**

Hip fractures in frail patients result in excess mortality not accounted for by age or comorbidities. The mechanisms behind the high risk of mortality remain undetermined but are hypothesized to be related to the inflammatory status of frail patients.

**Methods:**

In a prospective observational exploratory cohort study of hospitalized frail hip fracture patients, 92 inflammatory markers were tested in pre-operative serum samples and markers were tested against 6-month survival post-hip fracture surgery and incidence of acute kidney injury (AKI). After correcting for multiple testing, adjustments for comorbidities and demographics were performed on the statistically significant markers.

**Results:**

Of the 92 markers tested, circulating levels of fibroblast growth factor 23 (FGF-23) and interleukin-15 receptor alpha (IL15RA), both involved in renal disease, were significantly correlated with 6-month mortality (27.5% overall) after correcting for multiple testing. The incidence of postoperative AKI (25.4%) was strongly associated with 6-month mortality, odds ratio = 10.57; 95% CI [2.76–40.51], and with both markers plus estimated glomerular filtration rate (eGFR)– cystatin C (CYSC) but not eGFR-CRE. The effect of these markers on mortality was significantly mediated by their effect on postoperative AKI.

**Conclusion:**

High postoperative mortality in frail hip fracture patients is highly correlated with preoperative biomarkers of renal function in this pilot study. The effect of preoperative circulating levels of FGF-23, IL15RA, and eGFR-CYSC on 6-month mortality is in part mediated by their effect on postoperative AKI. Creatinine-derived preoperative renal function measures were very poorly correlated with postoperative outcomes in this group.

## 1 Introduction

Over 1.5 million patients worldwide suffer a hip fracture each year (1). According to the World Health Organization (WHO) hip fractures permanently disable 50% of sufferers and have a 1-year post-fracture mortality around 24% (2–4). The mortality rate after a hip surgery is significantly higher compared to the age and gender-matched population (5). Post-surgical mortality globally has remained mostly static over the past decade despite comprehensive best-practice surgery and rehabilitation packages (6–9). The overall incidence rates and costs of hip fractures are expected to rise (10), with estimated annual incidence rising to 6 million a year by 2050 worldwide (11) and costs estimated at 1.4% of total social and healthcare budgets.

Patients with a hip fracture are at substantial risk of major complications including cardiovascular, infectious, neurocognitive, and mortality (12). Several systematic reviews have found consistently that most of the associated factors for functional recovery and mortality of elderly hip fracture are biological, sociodemographic, or inherent to patients’ baseline characteristics (13–15).

A large proportion of frail individuals who suffer hip fractures have mild to severe impaired kidney function at the time of admission (16). Evidence-based approaches show that around 75% of hip fractures are frail, and preoperative frailty is associated with poorer outcomes and higher risk of postoperative mortality (17). Frailty is defined as a status of extreme vulnerability to stressors and is highly correlated with higher systemic inflammation (18). Importantly, the main causes of 12-month mortality for elderly hip fracture patients are cardiovascular disease and Alzheimer’s disease (19), both of which have inflammation as a central mechanism (20, 21).

Understanding the specific mechanisms involved in the increased risk of mortality associated with delayed surgery and the responsiveness of such mechanisms to surgery and rehabilitation are crucial to the development of interventions that improve outcomes from hip fracture surgery and reduce mortality and complications of fragility fractures. We have hypothesized that higher rates of mortality among individuals with pre-operative frailty might be driven by specific inflammatory pathways.

To explore this possibility, we recruited a pilot cohort of frail hip fracture patients from the busiest major trauma center in the United Kingdom. We compared their mortality, frailty levels, and basic characteristics to those from the same trauma center. Having obtained serum samples at the time of going into the operating theater, we carried out proteomic profiling and identified the molecular markers correlated with 6-month mortality, and assessed whether these were associated with incidence of postoperative acute kidney injury (AKI).

## 2 Methods

### 2.1 Study design

**Figure 1:**
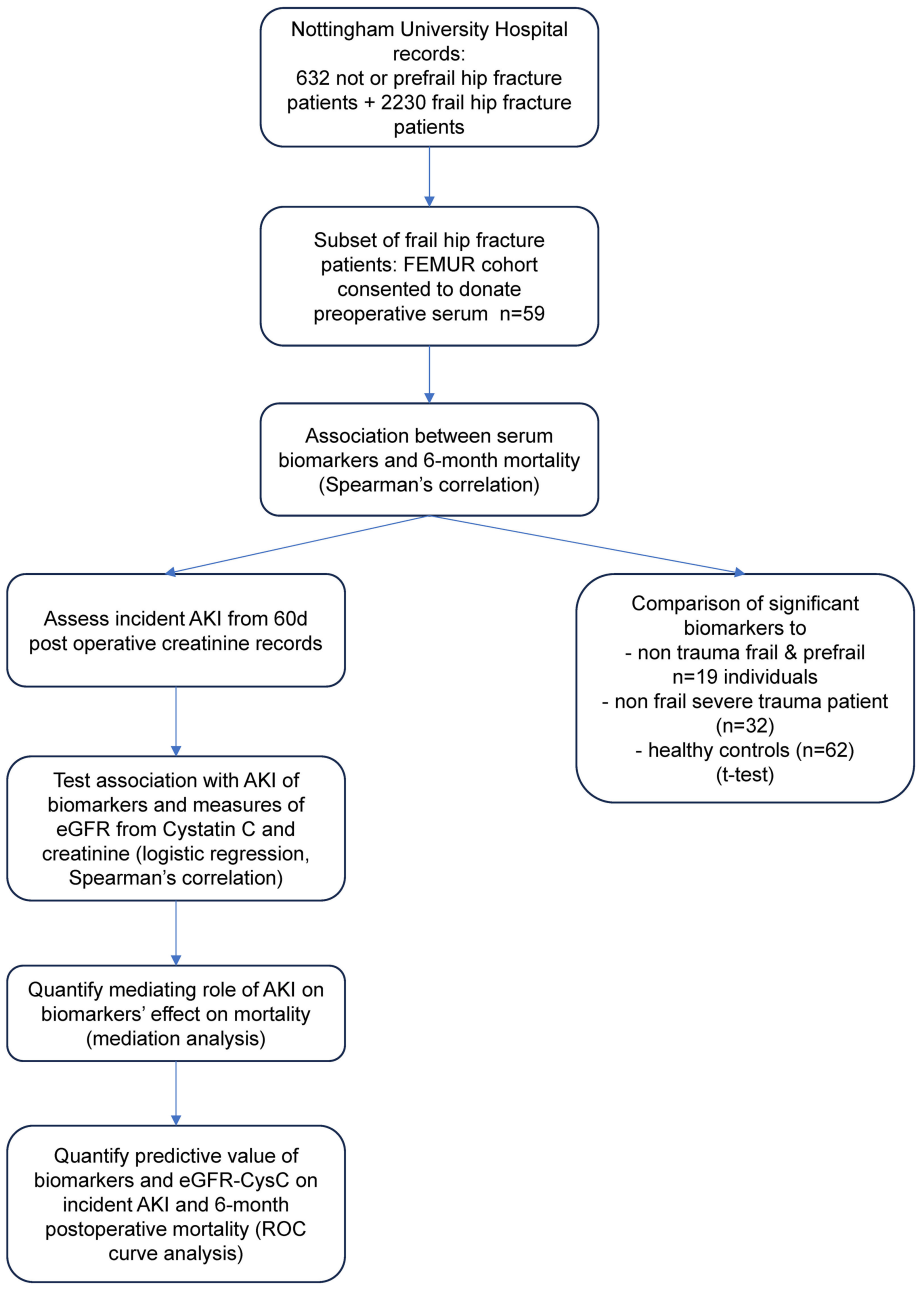
Flowchart of the study design.

A summary of the study design is presented in **Figure 1**. In this prospective, observational cohort study, serum preoperative samples were collected from a sub-cohort of frail hip fracture patients, with outcomes assessed at 6 months. Serum markers were tested as predictors of postoperative mortality and incident AKI in the sub-cohort. Levels of the significantly associated serum markers were also compared to positive and negative controls from additional comparator cohorts.

### 2.2 Participants

Fifty-nine frail hip fracture patients from the Functioning of Elder Muscle; Understanding Recovery (FEMUR) cohort were consented to give blood samples at induction of anesthesia (clinicaltrials.gov NCT04764617) (see **Table 1** for descriptive characteristics). This cohort recruited frail individuals admitted to hospital with a hip fracture. All participants were over 65 years of age, with a clinical frailty score of 4 or above and had suffered a hip fracture following a fall. All patients were recruited from Nottingham University Hospitals National Health Service (NHS) Trust. The CONSORT diagram for recruitment is shown in **Supplementary Figure S1**. The study protocol has been uploaded to be made publicly available at the University of Nottingham’s research data repository: https://www.nottingham.ac.uk/dts/researcher/managing-data/research-data-repository.aspx.

**Table 1:** Comparison between electronic health records (EHRs) for hip fracture surgeries at Nottingham University Hospitals in the same period and the enrolled sub-cohort.

	Hospital records				Recruited frail sub-cohort		*p*-value frail vs. non-frail EHRs	*p*-value frail EHR vs. FEMUR sub-cohort	
Frailty category	Not frail or prefrail		Frail		FEMUR				
***n***	632		2230		59				
	Mean	SD	Mean	SD	Mean	SD			
**Age years**	78.91	8.41	85.23	8.02	88.34	5.93	< 0.0001	0.003	
**Sex, F%**	66.93%		70.27%		84.75%		0.0875	0.019	
**Clinical frailty score (CFS)**	3.00	1.32	5.40	1.24	5.71	1.15	< 0.0001*	0.601	*
**Charlson’s comorbidities index** **(CCI)**	4.67	1.76	6.27	1.97	5.78	1.54	< 0.0001*	0.051	*
**NHFS-mortality**	5.25	2.96	8.91	5.30	8.20	4.79	< 0.0001*	0.107	*
**CKD %**	13.13%		28.21%		33.90%		< 0.0001*	0.558	*
**admission eGFR %**	73.92	17.01	64.23	20.56	61.85	19.70	< 0.0001*	0.998	*
**Length of stay (LOS) days**	12.99	8.46	18.02	12.87	19.48	13.07	< 0.0001*	0.299	*
** 180-day mortality**	6.33%		26.68%		27.12%		< 0.0001*	0.876	*
**Change in housing status**	21.99%		46.10%		40.68%		< 0.0001*	0.258	*

*p-value from logistic regression adjusted for age and sex.

Four comparator cohorts were assembled, including an established local registry that has been described elsewhere (22), which includes data collected from the electronic health records (EHRs) for all individuals who underwent a hip fracture surgery during the same period at Nottingham University Hospitals Trust. Patients were sequentially admitted neck of femur fracture patients between 01/01/2019 and 31/12/2022.

EHRs from Nottingham University Hospital’s Trust for individuals who underwent hip fracture surgery during the same period were collected. Patients in this cohort were sequentially admitted neck of femur fracture patients admitted between 01/01/2019 and 31/12/2022. This data was collected as part of an established local registry that has been described elsewhere.28

The PANdemic Tracking of HEalthcare woRkers (PANTHER) cohort involved asymptomatic healthcare workers who were assessed for SARS-CoV-2 infection during the first U.K. wave of the COVID-19 pandemic using symptom questionnaires and antibody testing. These participants donated blood to the Nottingham Tissue Bank for antibody testing and completed up to 16 weeks of symptom questionnaires. A randomly selected subset of this cohort has been included in this study as the negative control group. Sixty-two participants from the PANTHER cohort were randomly selected to be included in this study as the healthy control group, ensuring ethnic diversity of the sample. This study was initially under a Human Tissue Authority license in Nottingham (license number: 11035) and subsequently received ethical approval from North West, Greater Manchester South Research Ethics Committee, reference 20/NW/039 (registration number: NCT04318314). This group was included as a healthy control cohort.

The Orif Procedure mEchanisms of Rib Fixation (OPERA) cohort is an embedded mechanistic sub-study within the ORiF’s randomized controlled trial (RCT) aimed at investigating the mortality, quality of life, and cost effectiveness of operative rib fixation plus supportive management compared to supportive management only for patients admitted with three or more rib fractures. The OPERA sub-study that collects preoperative sera for these impact fracture patients has been included in this study as the positive control group. This group was included as a positive control of severe orthopedic trauma in the absence of frailty.

The prefrail sub-cohort included a subset of participants over 65 years of age who were recruited into an OA study and donated serum samples. Participants who were over 65 years and considered to be prefrail using the FRAIL scale combining the three items addressing Fatigue, Resistance (climbing steps), Ambulation (walking), together with weight loss and morbidity counts. Participants fulfilling one or two criteria may be classified as “pre-frail” and three or more as frail. This study received ethical approval from East Midlands Research Ethics Committee, reference 20/EM/0065 (registration number: NCT0442452). This group was used as a positive control of frailty in the absence of orthopedic trauma.

### 2.3 Exposures and outcomes

The key exposure was hip fracture. The primary outcome was 6-month postoperative mortality. The following comorbidities, confounder, and secondary outcomes were included: Charlson comorbidities index (CCI) (23) provides a measure of comorbid condition severity and has been widely validated, (24) was derived from patients’ hospital medical records. Specifically, the CCI includes myocardial infarction, congestive heart failure, peripheral vascular disease, cerebrovascular disease, dementia, chronic pulmonary disease, connective tissue disease, peptic ulcer disease, mild liver disease, diabetes mellitus (with or without complications), hemiplegia or paraplegia, renal disease, any malignancy (except malignant neoplasm of the skin), moderate or severe liver disease, metastatic solid tumors, and AIDS/HIV. Each condition is assigned a based on its severity and impact on mortality risk. The total score is calculated by summing up the scores for each comorbidity present in a patient, with higher scores indicating a higher risk of mortality. The presence of renal disease coded as chronic kidney disease (CKD) along with all the other comorbidities that are part of the CCI were also recorded at admission. The Rockwood Clinical Frailty Scale (CFS) is a judgment-based frailty tool that evaluates specific domains including comorbidity, function, and cognition to generate a frailty score ranging from 1 (very fit) to 9 (terminally ill) (25). The Nottingham Hip Fracture Score (NHFS) is a validated predictor of 30-day mortality in neck of femur fracture patients using demographic and clinical parameters recorded immediately preoperatively (26).

CFS, CCI, NHFS, age, sex, date and time of admission, surgery, and date of death (where applicable) were recorded for all FEMUR participants and were extracted from EHRs from the same hospital.

A measure of estimated glomerular filtration rate (eGFR) at admission, derived from serum creatinine, was available for all FEMUR study participants enrolled. eGFR immediately preoperatively (36.6h on average) from hospital records was available only for a subset; therefore, we obtained two additional measures of preoperative renal function eGFR from creatinine [CRE using CKD-EPI 2009 (27)] and cystatin C [CYSC, using CKD-EPI2012Scr-cys (28)]. Serum CYSC is a low-molecular-weight protein found in all tissues in the body, is filtered at the glomerulus and not secreted into the renal tubules or reabsorbed into the bloodstream, (29) and hence its use is recommended instead of creatinine to estimate GFR in adults with or at risk for CKD (30).

Incident AKI was assessed from creatinine recorded values for all FEMUR patients over the 2 months immediately post-operatively. Incident AKI was defined using the Kidney Disease: Improving Global Outcomes (KDIGO) criteria as either an increase in creatinine from baseline of ≥50% or an absolute rise of ≥27 micromol/l if within 48h (31).

### 2.4 Laboratory measurements

Serum was extracted from blood collected from all FEMUR patients at the time of going into the operating room. Venous blood was collected using a butterfly needle into 5 ml serum separation tubes containing clot activator and polymer gel. Tubes were left at room temperature for 20–30 min before being centrifuged for 5 min at 3000 RPM. The resulting serum was aliquoted into cryo-vials and stored immediately at −80. Inflammatory protein measurements were performed using a proximity extension assay method (Olink Bioscience, Uppsala, Sweden) (32) with the Olink inflammatory panel (33). ELISAs to measure serum levels of creatinine and cystatin C were performed by Affinity Biomarkers, London, using the Clinical Chemistry Analyzer. Preoperative eGFR was assessed from both creatinine and cystatin C levels measured from the same serum aliquot used to measure inflammatory markers (see 34).

### 2.5 Statistical analyses

We computed the Spearman’s correlation between 6-month mortality and 92 proteins from the Olink inflammatory panel. Results were adjusted for multiple testing using a False Discovery Rate correction (35). We compared results to an age-matched cohort of 62 healthy individuals without injury (negative controls) and a cohort of 32 younger patients with traumatic fracture from the OPERA cohort (positive controls) and 19 prefrail and frail individuals without trauma using unpaired *t*-tests. Effect size of associations with mortality for clinical parameters and significant biomarkers was further evaluated using logistic regression analyses.

Mediation analysis was performed to assess the mediating effect of incident AKI. For all models, we report the percentage causal mediation effect (ACME), the percentage direct effect (ADE). ACME represents the average size of the effect of the presurgical biomarkers on postoperative mortality that is mediated by incident AKI, while ADE represents the direct effect of biomarkers on mortality.

The statistical power to compute a significant correlation between inflammatory circulating markers and postoperative outcomes was computed using StatsDirect version 3.3.5.

Analyses were performed using Python 3.11.1 (libraries pyplot, numpy, and scipy), GraphPad Prism 10 and R 4.3.2 (library mediation).

## 3 Results

### 3.1 Descriptive characteristics and proteomic results

The descriptive characteristics of 59 frail fracture patients from the pilot study (FEMUR) who had serum samples taken immediately pre-operatively were compared to frail and non-frail hip fracture patients in the same hospital (**Table 1**). The FEMUR sub-cohort of frail patients with preoperative serum is broadly comparable to the overall patient population of frail hip fracture patients operated at this major trauma center between 2019 and 2022 (*n* = 2230). After adjustment for age and sex, there were no statistically significant differences between the pilot cohort and the overall frail hip fracture population in any of the clinical parameters including preoperative comorbidities and post-operative length of stay and mortality. On the other hand, as expected, we observed highly significant differences in all preoperative clinical parameters and post-operative outcomes between frail and non-frail hip fracture patients operated in the Queen’s Medical Centre in Nottingham. We therefore concluded that the frail fracture pilot sub-cohort was suitably representative of the mortality and comorbidities in the frail hip fracture population.

**Figure 2:**
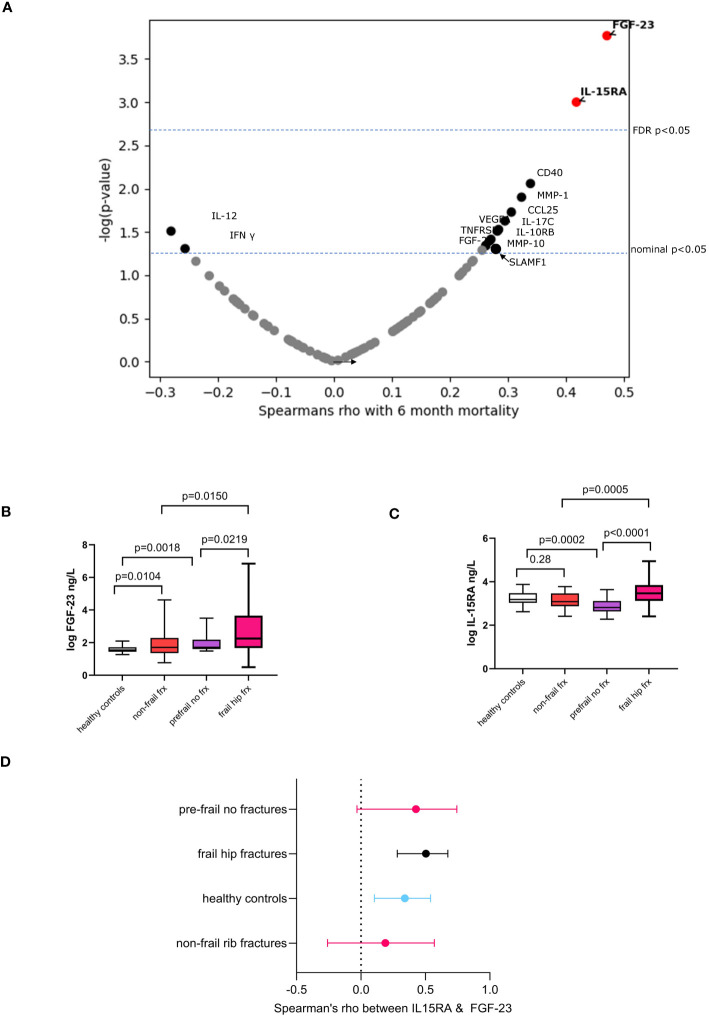
Associations for 6-month mortality and molecular inflammatory markers. **(A)** Volcano plot showing Spearman’s correlation coefficient and *p*-values. Boxplots showing the comparison of serum levels of **(B)** FGF-23 and **(C)** IL-15RA in frail hip fractures with regards to healthy controls, non-frail (impact) fracture cases and pre-frail individuals with no fractures. Box plots: mean values of molecular marker levels indicated by plus signs and medians indicated by bars in the boxes. The boxes show the interquartile range (IQR) and the whiskers extend from the boxes to indicate the 95% confidence intervals. **(D)** Forest plot showing the correlations between FGF-23 and IL-15RA in frail hip fracture patients and in the various comparator groups. Dots represent Spearman’s rho, whiskers are 95% confidence intervals.

In this sub-cohort, we then assessed the correlation between 92 inflammatory proteins and 6-month mortality. Thirteen proteins were nominally associated, namely, fibroblast growth factor 23 (FGF-23) and interleukin-15 receptor alpha (IL15RA), CD40, MMP-1, CCL25, IL-17C, VEGFA, IL-10RB, IL-12B, TNFRSF9, MMP-10, SLAMF1, and FGF-21. Of these, FGF-23 and IL15RA passed a multiple testing correction (**Figure 2A**). The minimum effect size needed to achieve 80% power with a *p* < 0.00054 (Bonferroni alpha = 0.05 for 92 independent tests) with the current sample size corresponds to correlation coefficient of 0.519 or higher. The strongest correlation observed in our study is 0.473 95% CI [0.25, 0.65] for FGF-23, which has a statistical power of 67.5%, that is, the present study was reasonably powered to detect the effect sizes actually observed.

We compared levels of these markers in frail fracture patients to *n* = 62 healthy controls (aged 42 ± 11.57 years), *n* = 30 non-frail multiple rib fracture patients (aged 60 ± 14.2 years, 9% female), and *n* = 19 frail/prefrail individuals with no fracture (age 70.05 ± 7.16 years) (descriptive statistics in **Supplementary Table S1**).

Levels of FGF-23 were increased in non-frail trauma compared to healthy controls and in pre-frail individuals without fractures compared to healthy controls (**Figure 2B**). In contrast, the pattern seen with IL-15RA shows a significant decrease in pre-frail versus healthy controls and no difference in non-frail trauma compared to controls (**Figure 2C**). Both markers have been implicated in kidney disease (36, 37). though with different mechanisms being involved for the role of these molecules in renal failure (38–40).

To better understand the relationship between FGF-23 and IL-15RA, we also assessed the correlations between the two markers in the various groups (**Figure 2D**) and find that they are in general very weakly correlated in healthy participants young and old and in individuals with severe orthopedic trauma but who are not frail. The relationship between both appears to be stronger in the frail fracture group that we studied here achieving a Spearman’s rho of 0.50 compared to the other patient and control groups analyzed.

### 3.2 Mortality, comorbidities and AKI

The presence of comorbidities was overall strongly associated with 6-month mortality (**Table 2**) with the odds ratio for each unit increase of the Charlson’s comorbidities index is 2.73, 95% CI [1.52–4.94] (**Table 2**).

**Table 2:** Association between clinical traits/biomarkers pre and post operative with 6-month mortality post hip fracture surgery in the FEMUR cohort.

Clinical feature/biomarker	Died within 180 d		Survived 180+ d		OR	95% CI	*p*-value
	Mean	SD	Mean	SD			
**Age**	89.71	4.79	86.90	6.16	1.11	0.98–1.27	0.09
**Sex (F %)**	88.2%		83.3%		1.50	0.28–8.08	0.64
**Clinical frailty score**	6.47	0.87	5.40	1.11	3.08	1.44–6.61	3.3E-04
**Nottingham hip frx score NHFS**	5.76	1.09	5.00	1.02	2.15	1.16–3.99	0.0127
**Charlson comorbidities index CCI**	7.06	1.14	5.40	1.18	2.73	1.52–4.94	3.3E-05
**Renal disease at admission (CKD)**	52.94%		23.81%		3.60	1.09–11.81	0.030
**Admission eGFR (from NHS records)**	50.76	22.59	69.02	22.60	0.95	0.92–0.98	0.004
**Preop eGFR CRE**	58.43	23.18	74.47	23.30	0.96	0.93–0.99	0.012
**Preop eGFR CYSC**	34.39	16.55	51.78	17.01	0.94	0.90–0.98	0.001
**Log FGF-23 ng/L**	3.79	1.68	2.37	1.74	1.80	1.21–2.67	1.7E-04
**Log IL-15RA ng/L**	3.85	0.48	3.39	0.50	4.90	1.52–15.81	0.001
**Incident AKI**	58.82%		11.91%		10.57	2.76–40.21	8.8E-05
**LOS (days)**	21.53	18.62	18.93	12.20	1.01	0.97–1.06	0.80

*p-value from logistic regression.

In addition, in this cohort, preoperative eGFR-CRE is only modestly associated with 6-month mortality, but eGFR-CYSC is more strongly associated (**Table 2**). Fifteen individuals (25.4%) developed AKI within the first 60 days after surgery, seven (11.8%) within 1-week postsurgery. Incidence of AKI was found to be strongly correlated with 6-month mortality (**Table 2**) corresponding to an odds ratio of 10.57, 95% CI [2.76–40.51].

We investigated the links between FGF-23 and IL15-RA, other markers of renal function, incident AKI, and 6-month post-surgical mortality. Overall, we find that of the 92 inflammatory markers measured preoperatively these are the two most strongly associated with post-operative incidence of AKI (**Supplementary Figure S2**).

**Figure 3:**
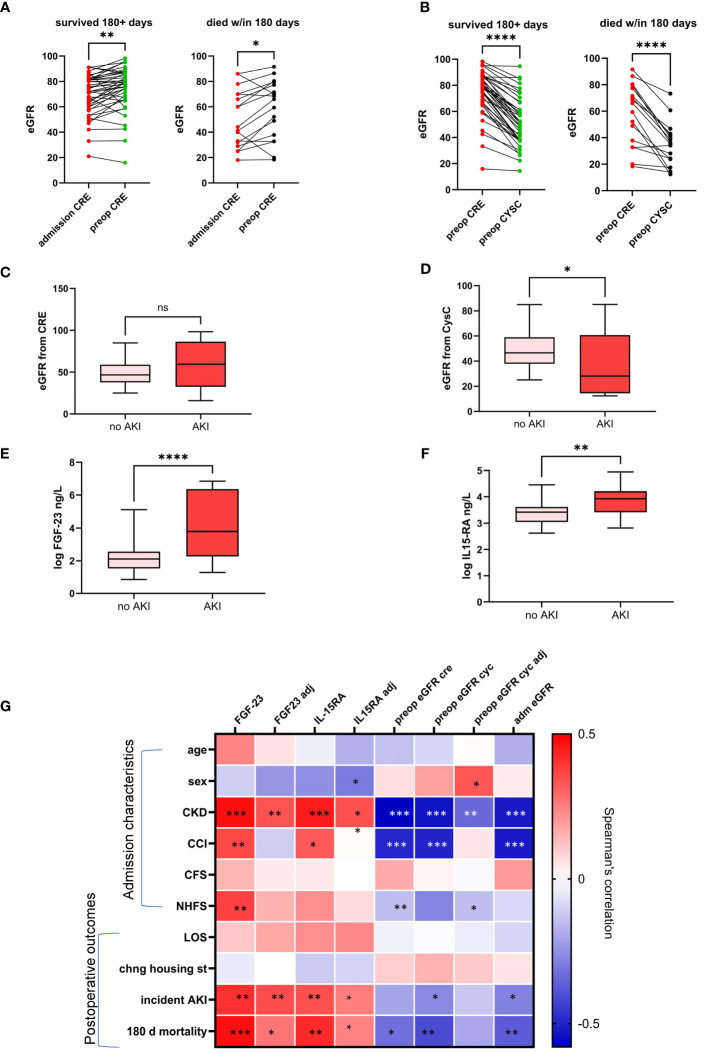
Change in eGFR from admission to immediately preoperatively measured from serum creatinine **(A)** and difference in eGFR preoperative estimates from serum creatinine and serum cystatin C levels **(B)**. Box plots showing the association between incident postoperative acute kidney injury (AKI) and preoperative eGFR from creatinine **(C)** and cystatin C **(D)** circulating IL15RA **(E)** and FGF-23 **(F)**. Heatmap showing correlations of admission characteristics and postoperative clinical outcomes with circulating biomarkers measured preoperatively with and without adjustment for age and comorbidities **(G)**. * p<0.05; ** p<0.01; *** p<0.001; **** p<0.0005; NS, not statistically significant.

A comparison of preoperative eGFR-CRE to admission values in hospital records showed that eGFR-CRE was significantly higher immediately preoperatively (**Figure 3A**). On the other hand, preoperative eGFR-CYSC was significantly lower compared to preoperative eGFR-CRE in both the survivor and deceased groups (**Figure 3B**). We then assessed the association between preoperative eGFR measures, FGF-23 and IL-15RA, and incidence of AKI. Preoperative eGFR-CRE was not significantly associated with postoperative incidence of AKI (**Figure 3D**) whereas preoperative FGF-23, IL-15RA, and eGFR-CYSC were all significantly correlated with incident AKI (**Figures 3E–G**).

The correlations between markers of renal function with and without adjustment for age and comorbidities on clinical measures at admission and surgical outcomes is summarized in a heatmap in **Figure 3G**. FGF-23 and IL-15RA remain associated with AKI after adjustment for age and comorbidities.

We also find that both markers are significantly associated with 6-month mortality after adjustment for age, sex and CCI (i.e., comorbidities). FGF-23 had an OR = 1.77, 95% CI [1.00–3.14] in units of standard deviations, and IL15-RA OR = 1.81, 95% CI [1.00–3.42] also in units of standard deviations).

**Figure 4:**
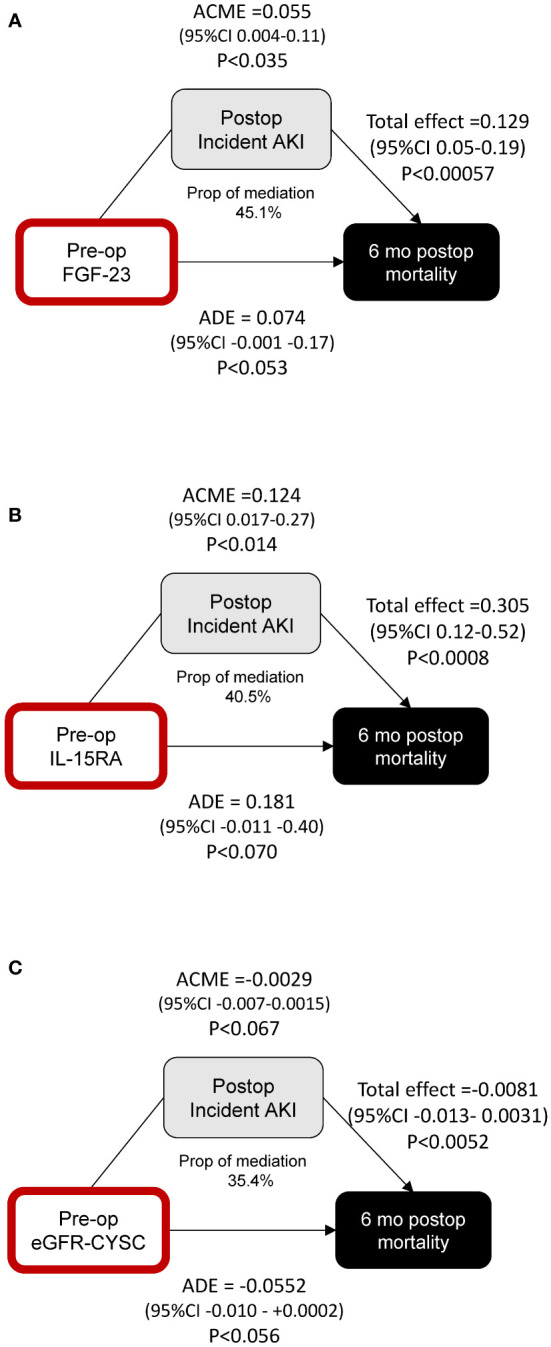
Mediation analysis of the effects of preoperative FGF-23 **(A)**, IL-15RA **(B)**, and eGFR-CYSC **(C)** on postoperative mortality, and the mediating effect of postoperative incident AKI. ROC curve analyses using clinical indices and biomarkers.

We performed formal mediation analyses to assess whether the effect of these markers on mortality was mediated by their effect on AKI. We find that the proportion of the effect of FGF-23 (**Figure 4A**) and IL-15RA (**Figure 4B**) on postoperative mortality mediated by incident postoperative AKI is statistically significant, with the direct effects not quite achieving statistical significance (*p* < 0.053 and *p* < 0.070). Neither the direct nor indirect effects alone for eGFR-CYSC achieved statistical significance (**Figure 4C**).

## 4 Discussion

In this exploratory pilot study, having tested 92 circulating inflammatory proteins, preoperative circulating levels of FGF-23 and IL-15RA were found to be significantly predictive of 6-month mortality post-surgery and of incident postoperative AKI. Having tested a large array of inflammatory markers, including various pro and anti-inflammatory cytokines and growth factors, it is notable that only proteins with links to renal function were predictive of postoperative mortality. This suggests that reducing the risk of AKI and its consequences should be a top priority in fragility fracture perioperative management which requires accurate preoperative renal function measurements. We also report that creatinine-based preoperative measures of renal function are only weakly associated with both outcomes whereas cystatin C-derived eGFR appears more informative and predictive.

Fibroblast growth factor–23 (FGF-23) is secreted by bone cells and is a key regulator of serum phosphate and active vitamin D3 levels (36). In late-stage CKD, FGF-23 cannot reduce serum phosphate levels, and high FGF-23 concentrations result in left ventricular hypertrophy (LVH), faster CKD progression, and mortality (41). FGF-23 is an independent predictor for LVH adjusting for eGFR (42, 43) and appears to be the molecular link between the bone’s physiological response to trauma (44, 45) and AKI (45) or an exacerbation of renal insufficiency in frail individuals (44). FGF-23 has also been identified as a predictor of AKI and death following cardiac surgery (46–48).

Two recent meta-analyses have found that FGF-23 can predict incident AKI in various patient populations in adults and children, combining data from critically ill patients, acute heart failure patients, patients with sepsis, and patients with acute respiratory distress syndrome (47, 48). FGF-23 has also been identified as a predictor of AKI and death following cardiac surgery (46–48). However, none of the studies had investigated the role of FGF-23 as a biomarker of AKI in hip fracture patients, even though post-operative incidence of AKI is known to risk factor of hip fracture mortality (49–53).

In addition to FGF-23, we also report for the first time that IL-15RA might also be a biomarker of post-operative incident AKI. IL-15RA mediates pleiotropic proinflammatory signals involved in several inflammatory and cardiovascular disorders (54). IL-15RA was identified (37) as one of 17 proteins involved in 10-year risk of end-stage renal disease in two large U.S. cohorts of individuals with type 2 diabetes. It is also one of 21 proteomic biomarkers associated both with kidney function decline and incident CKD over a 13-year period in a large longitudinal German study (55). IL-15, the natural ligand of IL-15RA is one of the key cytokines regulating the biology of natural killer (NK) cells (56–58).

There is now a substantial body of evidence showing that NK cells contribute to kidney injury and kidney failure by contributing to acute tubular necrosis in humans (57, 59, 60). Thus, it is likely that the effect of IL-15RA might be mediated by the role of its ligand on NK cells.

The data presented here show, for the first time to the best of our knowledge, that circulating levels of IL-15RA are linked to incidence of AKI. We also report the novel finding that this molecule is a biomarker of postoperative mortality in hip fracture patients.

Furthermore, we report very different patterns of these two markers in the comparator groups with, for example, IL-15RA being lower in pre-frail elderly individuals than in healthy controls, unlike the situation seen with FGF-23. 1L-15 has been shown to be reduced in ageing animal models and given that IL-1RA regulates the localization in immune cells of IL-15 (40, 61). This might explain why IL-15RA is lower in a pre-frail or frail individuals than in healthy controls. On the other hand FGF-23 increases with age and is in fact associated with incidence and prevalence of frailty in our data (62, 63).

In our data, eGFR-CYS is much more strongly associated with postoperative mortality than eGFR-CRE. The shortcomings of creatinine-based eGFR in frail individuals have been extensively described (29, 30, 64). As frail individuals lose muscle mass, a process accelerated by trauma, the amount of creatinine released from muscle into serum declines, resulting in over-estimation of eGFR and reduced accuracy for measurement of kidney function (34). The use of eGFR-CYSC is not affected by alterations in muscle mass and may offer advantages in such scenarios compared to eGFR-CRE (29, 30, 34, 64).

As already mentioned, AKI is a common complication in patients with hip fracture (51, 65, 66). Identifying and monitoring patients at increased risk may underpin strategies to improve outcomes. The value of targeting AKI in hip fracture patients has been recently demonstrated in an interventional study of withholding certain medications preoperatively in emergency and elective T&O patients (67). The investigators found a significant reduction in postoperative AKI (67).

Preoperative measures of eGFR based on creatinine levels appear poorly associated with mortality and incidence of AKI in the frail hip fracture patients in our cohort. This is consistent with various studies showing that eGFR-CYS is more reliable than eGFR-CRE in several patient populations with a variety of acute and chronic illnesses (68, 69). Our results, showing fairly poor performance of preoperative creatinine as a predictor of AKI, are therefore in agreement with what is widely accepted in the literature given the high levels of inflammation and of muscle loss seen in frail hip fracture patients.

Importantly, the strongest marker of incident AKI in our study was FGF-23, followed by IL-15RA. We show here that a substantial proportion of the effect of these biomarkers is mediated by the postoperative incidence of AKI. If these biomarkers were validated in larger study samples, this would be a strong incentive to incorporate such measures within clinical practice to improve patient care. We also find that, along with FGF-23 and IL-15RA, eGFR of cystatin C is also much more informative of AKI incidence than creatinine derived eGFR. Although cystatin C has already been assigned more significant role in estimating glomerular filtration rate (GFR) in the recent KDIGO guidelines (70), this marker is up to 10 times as expensive as creatinine, which might explain why its use has not been more widely adopted in various clinical settings (71). Future studies should focus on defining the health economic benefits of obtaining more reliable predictors of post-operative AKI incidence in this patient population and on exploring how to improve clinical management to reduce the incidence of AKI and therefore also post-operative mortality.

The current study has several strengths. The pilot sub-cohort analyzed for biomarkers is comparable in clinical and demographic characteristics to a much larger cohort of frail hip fracture patients from the largest trauma centre in the United Kingdom, suggesting that the data from our study should be generalizable to the wider hip fracture populations. We were able to compare the biomarkers tested to a series of relevant positive and negative controls, namely healthy controls, non-frail severe fractures, and non-fractured prefrail and frail individuals. Through the analysis of EHRs, we were also able to compare the predictive value of biomarkers for mortality to that of combined clinical and demographic indices fitted in the large EHR database. Overall, the conclusions of the study, namely, that biomarkers of renal failure and AKI are the most highly associated with post-operative mortality after hip fracture surgery, are consistent with a large body of literature showing the role of these biomarkers in renal disease and of kidney injury in post-surgical mortality.

The key limitation of the present study is the pilot nature of the data, that is, the sample size of fracture cases with preoperative serum. The small sample size also means that there may be other preoperative inflammatory markers in the 92-protein panel that are associated with post-operative mortality but these are not detected as statistically significant after adjusting for multiple tests due to the statistical power available in this study. On the other hand, some of the limitations of a small sample size, specifically lack of representativeness, have been compensated for by ensuring that key clinical parameters are comparable to those in the larger frail hip fracture patient population from the largest Major Trauma Centre in England. Furthermore, we note that the study was sufficiently powered to detect the size effects that were actually measured and that there is strong face validity for the two markers identified given their links to kidney injury.

In conclusion, an exploratory study investigating inflammatory mediators of high-postoperative mortality in hip fracture patients identified renal-related factors as strong predictors of mortality. Our data suggest that either FGF-23 or IL-15RA or both should be assessed as potential biomarkers to make accurate clinical decisions regarding risk of postoperative AKI and to evaluate the risk of mortality and complications in frail hip fracture patients.

## Data availability statement

The original contributions presented in the study are included in the article/**Supplementary Material**, further inquiries can be directed to the corresponding author/s.

## Ethics statement

The studies involving humans were approved by Camden and Kings Cross Research Ethics Committee. The studies were conducted in accordance with the local legislation and institutional requirements. The participants provided their written informed consent to participate in this study.

## Author contributions

AVa: Writing – original draft, Writing – review & editing, Conceptualization, Formal analysis, Funding acquisition, Investigation, Methodology, Project administration, Supervision. AI: Conceptualization, Funding acquisition, Investigation, Writing – original draft, Writing – review & editing, Data curation. LT: Writing – original draft, Writing – review & editing. AZ: Data curation, Writing – original draft, Writing – review & editing. AKo: Writing – original draft, Writing – review & editing. AKe: Writing – original draft, Writing – review & editing. WA: Writing – original draft, Writing – review & editing. AVi: Writing – original draft, Writing – review & editing. SM: Writing – original draft, Writing – review & editing. JN: Writing – original draft, Writing – review & editing. NS: Writing – original draft, Writing – review & editing. BO: Funding acquisition, Writing – original draft, Writing – review & editing.
