# Supplementary material for: Preoperative inflammatory biomarkers reveal renal involvement in postsurgical mortality in hip fracture patients: an exploratory study
Source: Front Immunol. 2024 Jun 10;15:1372079. doi: 10.3389/fimmu.2024.1372079 (PMC11197399; doi:10.3389/fimmu.2024.1372079)
Supplement: Supplementary file 1 [file DataSheet_1.docx]

Supplementary Material

# Supplementary Table 1

**Supplementary Table 1** Descriptive characteristics of hip fracture (FEMUR) cohort, health controls (PANTHER) and multiple rib fractures (OPERA) cohort.

|  |  | Hip fractures (frail) (n=59) | Healthy controls (n= 62) | Multiple rib fractures (non-frail) (n= 30) | Prefrail controls (n=12) |
| --- | --- | --- | --- | --- | --- |
| Age | Mean years | 87.7 | 42 | 60.4 | 70.1 |
|  | SD | 5.9 | 11.57 | 14.2 | 7.16 |
| Sex | M | 9 (15%) | 16 (26%) | 21 (70%) | 3 (26%) |
|  | F | 50 (85%) | 46 (74%) | 9 (30%) | 9 (74%) |

**Supplementary Figures**

**Supplementary figure 1.** CONSORT flowchart of FEMUR cohort through study.

**Supplementary Figure 2.** Correlation (Spearman’s rho) between 92 inflammatory markers from the Olink panel and incident AKI in the FEMUR cohort.

**
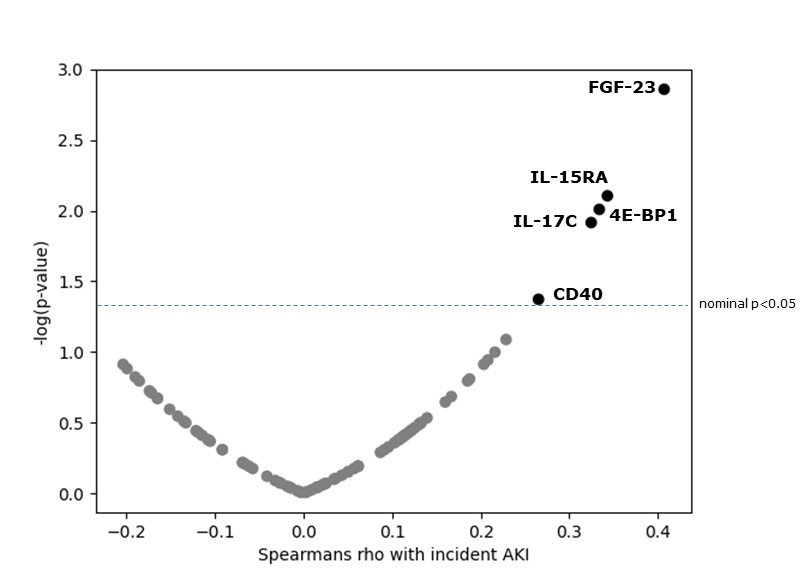
**
